# Prevalence and psychosocial correlates of depressive symptom risk among secondary school students in Saudi Arabia: a mixed-methods study

**DOI:** 10.3389/frcha.2026.1926540

**Published:** 2026-09-02

**Authors:** Ibrahim Awad Eljack Ibrahim, Abdullah M. Alshahrani, Abdullah Hassan Alhalafi, Kamal Eldin Hussein Elhassan, Abubakar Mohamed Jibo, Partha Nandi, Adel M. Alshahrani, Ahmed A. Alghamdy, Awn S. Alhersh, Ayed M. Al-Qahtani, Asim M. Alasmari, Ali H. Alhelal, Hani Abdulrahman F. Al-Qahtani, Hassan S. Alshahrani, Ribi M. Alhersh

**Affiliations:** 1 Department of Family and Community Medicine, College of Medicine, University of Bisha, Bisha, Saudi Arabia; 2 College of Medicine, University of Bisha, Bisha, Saudi Arabia

**Keywords:** adolescent depression, bullying, mental health, mixed-methods study, psychosocial risk factors, Saudi Arabia, school mental health, secondary school students

## Abstract

**Objectives:**

Adolescent depression is a major public health concern with important psychosocial and educational implications. This study examined the distribution of a depressive-symptom-risk/general-distress composite and its psychosocial correlates among secondary school students in Bisha Province, Saudi Arabia, and explored teachers' perspectives on how depressive symptoms or distress affect students in school settings.

**Methods:**

A school-based cross-sectional mixed-methods study was conducted during the 2024–2025 academic year. Quantitative data were analyzed from 425 secondary school students using a self-administered questionnaire containing a 17-item depressive-symptom-risk composite and psychosocial stressor items. Composite items were coded from 0 to 7 (theoretical total range 0–119; observed range 0–84). For descriptive purposes, four sample-specific, non-diagnostic score bands were used (0–24, 25–38, 39–67, and ≥68), and the primary logistic-regression outcome was dichotomized as 0–24 vs. ≥25. Analyses included chi-square tests, Spearman correlation, Kruskal–Wallis tests, and multivariable logistic regression, with *p*-values corrected for multiple comparisons using the Benjamini–Hochberg procedure. Qualitative data were obtained through semi-structured interviews with 42 teachers and analyzed thematically. Ethical approval was obtained from the University of Bisha Institutional Review Board.

**Results:**

Overall, 47.1% of students had an elevated composite score (≥25). In the 425-student analytical sample, family conflict (aOR = 5.47) and emotional or physical violence (aOR = 3.40) remained significantly associated with elevated scores after correction for multiple comparisons; bullying showed a positive uncorrected association (aOR = 1.68) that did not survive correction. Health-related conditions, smoking, and family history of mental illness were also associated with higher score bands in bivariate analyses. Gender-stratified analyses showed that, after correction, family conflict remained significant among male students, while family conflict and emotional/physical violence remained significant among female students; associations for bullying among males and sexual harassment among females were positive but did not survive correction and should be interpreted as suggestive. Composite scores were not significantly associated with academic performance (CGPA). Teachers reported emotional withdrawal, behavioral changes, reduced classroom engagement, and limited training in identifying and supporting affected students.

**Conclusions:**

Elevated depressive-symptom-risk composite scores were common among secondary school students and were strongly associated with modifiable psychosocial stressors, particularly family conflict and exposure to violence. Because the instrument and its score bands are not diagnostic thresholds, the findings should be interpreted as relative psychosocial/depressive-symptom risk rather than prevalence of clinical depressive disorder. Given the cross-sectional design, these associations are correlational rather than causal. Findings highlight the need for validated school-based mental health screening, teacher training, and family-centered interventions.

## 1 Introduction

Depression among adolescents is a growing public health concern worldwide, with far-reaching implications for mental health, academic performance, and social development. Adolescence is a critical developmental stage characterized by rapid biological, psychological, and social transitions that heighten vulnerability to mental health disorders (1). The World Health Organization estimates that up to 20% of adolescents experience a mental health condition, with depression among the most common (1). In school contexts, depression can interfere with concentration, motivation, participation, and attendance, reflecting how psychosocial distress translates into functional academic and behavioral challenges (2, 3). When persistent and untreated, adolescent depressive disorders are also associated with substance use, impaired social and later occupational functioning, suicidal behavior, and persistence of depressive illness into adulthood (4).

For the purposes of this study, depression is conceptualized along a continuum ranging from subclinical depressive symptoms to major depressive disorder as defined in DSM-5, and is characterized by core affective features — persistent low mood (dysphoria) and loss of interest or pleasure (anhedonia) — together with associated cognitive, somatic, and behavioral symptoms such as sleep and appetite disturbance, fatigue, poor concentration, feelings of worthlessness, and suicidal ideation. The present study uses a self-report screening instrument to estimate depressive symptom risk and burden in a general school-based population; it does not constitute, and should not be interpreted as, a clinical diagnosis. This distinction is described further in Section 2.4 and revisited in the Limitations.

A growing body of international research demonstrates a strong relationship between depression and educational outcomes. Wei et al. showed that concentration problems play a mediating role between depression and problematic smartphone use, underscoring how cognitive symptoms of depression impair academic functioning (2). Similarly, a study of South African undergraduates found that depressive symptoms were negatively correlated with academic performance, reflecting the broad impact of mental health on educational success (5). In China, longitudinal evidence during the COVID-19 pandemic highlighted that poor sleep quality and anxiety symptoms were significantly associated with depression, which was in turn associated with reduced classroom engagement (6). Beyond acute effects, long-term studies suggest a bidirectional relationship: depression is associated with lower achievement, while persistent academic struggles may reinforce depressive symptoms, forming a mutually reinforcing cycle (7). This association is not limited to students; a large cross-sectional study of English secondary school teachers found that depressive symptoms were associated with work dissatisfaction, feeling unable to seek support from colleagues, and lower student attendance, further illustrating how teacher mental health and the classroom environment are interlinked (8).

These patterns are not confined to Asia and Africa. Population-based surveys in North America likewise document substantial adolescent depression burden: the 2024 U.S. National Survey on Drug Use and Health estimated that 15.4% of adolescents aged 12–17 had experienced a past-year major depressive episode (9), while Canadian survey data indicate that approximately 7% of youth report past-year depression, within a global range of roughly 3% to over 10% across pediatric populations (10). This indicates that adolescent depression is a global phenomenon rather than one confined to any single region.

Evidence from low- and middle-income countries adds further urgency. A Nigerian study demonstrated that insecurity and psychosocial stressors were significantly associated with depression, which in turn was associated with adverse academic outcomes (11). These findings resonate with challenges faced by students in the Middle East, where cultural, familial, and educational pressures shape adolescent experiences in unique ways. In Saudi Arabia, recent national survey data suggest that approximately 40% of youth aged 15–30 years have experienced a mental disorder, with mood disorders such as depression particularly common, yet only a minority receive treatment (12).

Local studies also indicate substantial rates of adolescent depression. AlYousefi et al. found that 32.4% of intermediate- and secondary-school students in Riyadh had moderate-to-severe depression on the PHQ-9 (13). In the Qassim region, Alharbi et al. reported 34.0% mild, 24.6% moderate, 10.4% moderately severe, and 5.0% severe depression among high-school students (14). Among secondary-school girls in Buraydah, Alenzi and Suliaman documented a depression prevalence of 21.6%, with many affected students falling in the moderately severe or severe categories (15). These studies emphasize that reported prevalence varies substantially by population and assessment method.

A substantial body of developmental research has examined how proximal psychosocial stressors shape adolescent depressive symptoms. Family-level factors — including parental conflict, harsh or inconsistent discipline, and low family cohesion — have been consistently linked to elevated depressive symptoms; a recent meta-analysis of 46 studies confirmed a significant positive association between parent-adolescent conflict and depressive mood, consistent with attachment and emotional-security theories linking family relationship quality to adolescent emotional development (16). Peer-level stressors, particularly bullying victimization, contribute to a wide range of adverse mental-health outcomes; a systematic review and meta-analysis of 165 studies found the strongest evidence for causal associations between bullying victimization and depression, anxiety, and suicidal ideation and behaviors, with proposed mechanisms including social isolation, humiliation, and diminished self-worth (17). School-level pressures such as academic performance expectations and limited teacher support, and broader societal factors such as stigma around help-seeking and restrictive gender norms, may further compound adolescent vulnerability, as documented in regional reviews of adolescent mental health in the Arab world (18, 43). However, comparatively little is known about how these factors interact, or which are most salient, within Gulf and Middle Eastern cultural contexts, where family structure, gender norms, and stigma differ substantially from Western settings — a gap this study seeks to address.

Despite growing evidence of adolescent depression in Saudi Arabia, to the best of our knowledge, no study to date has simultaneously examined psychosocial correlates, gender-specific risk profiles, and school-level perspectives within a single convergent mixed-methods design. In particular, the role of frontline teachers in identifying and responding to student depression or distress — and the institutional barriers they face in doing so — remains largely unexplored in the Saudi context. Existing studies have also relied predominantly on quantitative screening tools without integrating the observational knowledge of educators who interact with students daily. This study therefore aimed to: (1) describe the distribution of the study's depressive-symptom-risk/general-distress composite among secondary school students in Bisha Province, Saudi Arabia; (2) identify psychosocial factors associated with an elevated composite score (≥25), including gender-stratified analyses; and (3) explore teachers' perceptions of how depressive symptoms or distress manifest and are managed within school settings. A convergent mixed-methods design was employed to provide a more complete and actionable understanding of adolescent psychosocial/depressive-symptom risk than either approach could offer in isolation.

## 2 Methodology

### 2.1 Study design and setting

This study employed a convergent mixed-methods cross-sectional design. Quantitative and qualitative data were collected during the same period and integrated during interpretation to provide a comprehensive understanding of the study's depressive-symptom-risk/general-distress composite and its psychosocial correlates among secondary school students in Bisha Province, Saudi Arabia. The study additionally examined the association between composite score and academic performance as measured by cumulative grade point average (CGPA). Bisha Province is in the southern part of the Kingdom, about 260 km north of Abha, and includes both urban and rural communities. The province has 46 secondary schools, 20 for boys and 26 for girls, together enrolling about 9,368 students aged 15–18 (4,709 boys and 4,659 girls).

### 2.2 Study population

The study population included male and female students attending public secondary schools in Bisha Province during the 2024–2025 school year. Teachers in these schools also participated in the qualitative component. Inclusion criteria for students were: (1) enrollment in Grade 10, 11, or 12 at a participating public secondary school in Bisha Province during the study period; (2) sufficient literacy to complete the Arabic-language questionnaire independently; and (3) provision of adolescent assent together with parental/guardian consent. Exclusion criteria for students were: (1) enrollment in schools serving students with intellectual or developmental disabilities, for whom the self-report format was not considered appropriate; and (2) incomplete or missing consent documentation. Teachers were eligible if currently employed at a participating school and willing to provide informed consent for a face-to-face interview; no additional exclusion criteria were applied to teachers.

Secondary education in Saudi Arabia spans three years (Grades 10–12) and typically enrolls students aged 15–18 years, with students generally around 15–16 years old in Grade 10, 16–17 in Grade 11, and 17–18 in Grade 12. The overall sample age range (13–19 years, median 17) reflects normal variation around these grade-level bands together with a small number of atypical enrollment ages.

### 2.3 Sampling and sample size

A stratified multistage cluster sampling method was used to ensure the sample represented the whole population. In the first stage, 8 schools were randomly selected from the 46 schools in the province (4 boys' schools and 4 girls' schools). In the second stage, one class was randomly selected from each grade level: 10th, 11th, and 12th. In the final stage, 16 students were randomly selected from each class, yielding a minimum target sample of 384 students. Although 384 participants were required, 426 completed questionnaires were obtained; following data screening, one participant with an extreme age value (25 years) was excluded, resulting in a final analytical sample of 425 students.

The minimum required sample size was calculated using Cochran's single-proportion formula, *n* = Z^2^p(1−p)/d^2^, where *Z* = 1.96 (95% confidence), *p* = 0.50 (a conservative value chosen to maximize the required sample size in the absence of a directly comparable validated prevalence estimate for this composite), and *d* = 0.05. This yielded *n* = 384.16, or approximately 384 students; the final analytical sample of 425 exceeded this target.

### 2.4 Data collection tools

Quantitative data were collected using a structured questionnaire comprising demographic characteristics, psychosocial risk factors, and a 17-item depressive-symptom-risk composite. Each composite item used an eight-level ordered response scale coded from 0 to 7, anchored at 0 (“Not at all”) and 7 (“Always”), with intermediate responses coded 1–6. The theoretical total score therefore ranges from 0 to 119; in the final analytical sample, observed scores ranged from 0 to 84. Higher scores indicate greater burden on the behaviors, somatic complaints, emotional-regulation difficulties, and distress indicators represented by the composite. Internal consistency in the 425-student analytical sample was good (Cronbach's *α* = 0.838).

The 17 items span emotional suppression/avoidance (e.g., “I ignored bad emotions,” “I hid my problems”), somatic complaints (headache, stomach pain, heartburn, unusual aches), aggression/irritability, impulsive or risk-taking behavior (including reckless driving and smoking-related behaviors), feeling unsupported, and difficulty controlling emotions. One item refers to attempting to ignore feeling depressed, but this assesses an avoidance response rather than directly measuring persistent dysphoria. No item directly assesses both DSM-5 core affective symptoms of persistent low mood and anhedonia, nor does the composite assess sleep, appetite, or suicidal ideation. Accordingly, throughout this manuscript the measure is interpreted as a depressive-symptom-risk/general psychosocial-distress composite rather than as a diagnostic depression scale. The complete 17-item wording is reported in Table 1.

**Table 1:** Item wording, descriptive statistics, and corrected item–total correlations for the 17-item composite.

No.	Composite item	Mean	SD	Corrected item–total r
1	I ignored bad emotions	3.49	2.82	0.321
2	I hid my problems	3.90	2.89	0.519
3	I smoke more than usual	0.18	0.98	0.348
4	I drove recklessly or violently	0.86	1.83	0.301
5	My heartburn was more than average	1.25	2.01	0.498
6	I had regular headaches	1.93	2.37	0.506
7	My stomach hurts	1.88	2.43	0.482
8	I was left to figure things out on my own	2.23	2.62	0.541
9	I experienced strange aches and sensations	1.96	2.54	0.654
10	I need to smoke to help me to relaxed	0.25	1.12	0.390
11	I need to have easy access to cigarettes	0.27	1.20	0.311
12	I reacted excessively and aggressively to circumstances	1.21	1.96	0.495
13	I stopped worrying about the results of my conduct	1.63	2.37	0.254
14	I took unnecessary dangers	1.20	2.08	0.574
15	I attempted to ignore feeling depressed.	0.90	1.82	0.451
16	I was verbally aggressive against others	0.72	1.61	0.451
17	It was tough to control my emotions	1.85	2.39	0.518

Items were coded 0–7. Cronbach's *α* = 0.838. Theoretical total-score range=0–119; observed analytical-sample range=0–84.

As a concrete example of data conversion, the endpoint response “0 Not at all” was coded as 0 and “7 Always” was coded as 7; intermediate response options retained their numeric values from 1 to 6. Thus, for the item “I ignored bad emotions,” a response recorded as “7 Always” contributed 7 points to the composite total, whereas “0 Not at all” contributed 0 points. This same coding rule was applied consistently to all 17 composite items.

For descriptive reporting, four score bands were retained from the original analysis: low (0–24), moderate (25–38), high (39–67), and extreme (≥68). In the 425-student analytical sample, these bands contained 52.9%, 24.2%, 19.8%, and 3.1% of students, respectively, and therefore describe the empirical distribution rather than validated clinical severity. The available dataset confirms these operational boundaries but does not document an external validation or a prospectively specified derivation for the cut-points; accordingly, no such validation is claimed. The theoretical upper bound of the composite is 119, although the highest observed score was 84. These cut-points are not PHQ-9, CES-D, DSM-5, or other diagnostic thresholds. For the primary multivariable analysis, scores were dichotomized as 0–24 (reference) vs. ≥25 (elevated score), separating the lower approximately half of the observed distribution from the remainder for an interpretable binary contrast; this dichotomy should not be interpreted as clinical case status.

The survey also assessed demographic and health characteristics and six psychosocial exposures analyzed: bullying, sexual harassment, emotional or physical violence, loss of a loved one, family conflict, and dissatisfaction with physical appearance. These exposure items were recorded as yes/no variables. Health-related conditions, family history of mental illness/depression, and smoking status were also recorded and examined as bivariate correlates.

Qualitative data were gathered through semi-structured interviews with teachers from the same eight participating schools. Teacher participation was voluntary and non-probability-based among eligible staff who consented to a face-to-face interview. Questions explored teachers' perceptions of student depressive symptoms or distress, behavioral and academic manifestations, challenges in recognition and support, and available or recommended support systems.

### 2.5 Data collection procedure

The questionnaire was distributed electronically via Google Forms and disseminated through school administrators to students' WhatsApp groups. Participation was voluntary and anonymous. Teachers were invited to participate in face-to-face interviews conducted in Arabic by trained research assistants, with consent recorded and the interviews transcribed for analysis.

### 2.6 Data analysis

Quantitative data were exported to Excel and analyzed using IBM SPSS version 26. Descriptive statistics were presented as frequencies, percentages, means, and standard deviations.

The normality of continuous variables was assessed using the Shapiro–Wilk test. Age (*W* = 0.892, *p* < 0.001) and CGPA (*W* = 0.603, *p* < 0.001) were not normally distributed; non-parametric methods were therefore used for analyses involving these variables.

Prior to analysis, data were screened for completeness and plausibility. One participant with an age value of 25 years was identified as an extreme outlier relative to the target adolescent population and was excluded from the final analysis.

Associations between categorical characteristics and the four composite-score bands were examined using chi-square tests of independence; age was compared across score bands using the Kruskal–Wallis test because of its non-normal distribution. Spearman's rho was used to assess the relationship between composite score and CGPA, and the Kruskal–Wallis test was used to compare CGPA across score bands. Benjamini–Hochberg correction was applied within related families of multiple tests (sociodemographic/health comparisons, psychosocial comparisons, and the six coefficients within each regression model). A corrected *p*-value < 0.05 was considered statistically significant.

Qualitative data were analyzed using thematic analysis. Three researchers independently coded all transcripts, followed by consensus meetings to reconcile discrepancies and finalize themes. Saturation was operationalized as the point at which successive interviews no longer generated substantively new codes or themes; later interviews were retained to confirm and refine the five-theme structure reported in Section 3.6. The exact interview number at which saturation was first judged was not prospectively recorded, so no *post hoc* saturation number is asserted.

The primary multivariable binary logistic regression examined the six psychosocial exposures of substantive interest—bullying, sexual harassment, emotional/physical violence, loss of a loved one, family conflict, and dissatisfaction with physical appearance—in relation to an elevated composite score (≥25 vs. 0–24). The same six-variable specification was used for the overall and gender-stratified models. Sociodemographic and health correlates were evaluated separately in bivariate analyses and were not part of this primary psychosocial model. Adjusted odds ratios (aORs) and 95% confidence intervals (CIs) were calculated. Multicollinearity was assessed using variance inflation factors; in the 425-student overall model, VIFs ranged from 1.20 to 1.80, indicating no problematic collinearity. Regression *p*-values were corrected across the six psychosocial coefficients using the Benjamini–Hochberg procedure.

### 2.7 Ethical considerations

Ethical approval was obtained from the Institutional Review Board (IRB) of the University of Bisha College of Medicine (Approval No. UBCOM/IRB/2023). Written informed consent was obtained from all participating students and teachers. For students under 18, parental consent was also secured. Confidentiality and anonymity were maintained throughout the study, and participants were informed of their right to withdraw at any time without consequences.

## 3 Results

### 3.1 Participant characteristics

A total of 425 secondary-school students were included in the quantitative analysis after exclusion of the 25-year-old outlier. The median age was 17 years (IQR = 2), with ages ranging from 13 to 19 years. Slightly more than half were female (51.8%). Most students lived with their families (87.5%), were not working (95.1%), and resided in Bisha city (78.1%). Approximately 29.6% reported at least one health-related condition, 7.8% reported a family history of mental illness or depression, and 5.6% were current smokers (Table 2).

**Table 2:** Participant characteristics of the analytical sample (*N* = 425).

Characteristic	Category/summary	*n* (%) or median (IQR)
Age, years	Median (IQR); range	17 (2); 13–19
CGPA, %	Median (IQR); range	99 (4); 39–100
Gender	Male	205 (48.2%)
	Female	220 (51.8%)
Living status	With family	372 (87.5%)
	Other	53 (12.5%)
Residence	Bisha city	332 (78.1%)
	Rural village	93 (21.9%)
Grade	Year 1	130 (30.6%)
	Year 2	156 (36.7%)
	Year 3	139 (32.7%)
Work status	Not working	404 (95.1%)
	Working	21 (4.9%)
Health-related condition	Yes	126 (29.6%)
Family history of mental illness/depression	Yes	33 (7.8%)
Current smoking	Yes	24 (5.6%)

One respondent aged 25 years was excluded before analysis. Percentages are based on *N* = 425.

### 3.2 Distribution of composite scores

Composite scores ranged from 0 to 84 in the analytical sample, with a mean of 25.7 (SD = 19.1). The low, moderate, high, and extreme sample-specific score bands contained 52.9%, 24.2%, 19.8%, and 3.1% of students, respectively. Using the binary contrast, 47.1% of students had an elevated score (≥25) (Table 3).

**Table 3:** Distribution of sample-specific composite score bands.

Score band	Operational boundary	*n*	%
Low	0–24	225	52.9
Moderate	25–38	103	24.2
High	39–67	84	19.8
Extreme	≥68 (observed 68–84)	13	3.1

These are empirical descriptive bands, not validated diagnostic or clinical-severity thresholds. Scores ≥25 were classified as elevated for the primary binary logistic-regression contrast.

### 3.3 Sociodemographic differences across composite score bands

Score-band distributions differed significantly by living status, health-related conditions, family history of mental illness/depression, and smoking status after Benjamini–Hochberg correction. No significant corrected differences were observed for age, gender, place of residence, grade level, or work status (Table 4).

**Table 4:** Sociodemographic and health characteristics across composite score bands.

Variable/level	Low *n* (%)	Moderate *n* (%)	High *n* (%)	Extreme *n* (%)	Uncorrected *p*	BH-adjusted *p*
Age, median (IQR)	17 (1)	17 (1)	17 (2)	17 (2)	0.611	0.785
Gender: female	123 (55.9%)	46 (20.9%)	44 (20.0%)	7 (3.2%)	0.411	0.617
Gender: male	102 (49.8%)	57 (27.8%)	40 (19.5%)	6 (2.9%)		
Living status: Other	19 (35.8%)	14 (26.4%)	19 (35.8%)	1 (1.9%)	0.009	0.019
Living status: With family	206 (55.4%)	89 (23.9%)	65 (17.5%)	12 (3.2%)		
Residence: Bisha city	174 (52.4%)	83 (25.0%)	66 (19.9%)	9 (2.7%)	0.787	0.886
Residence: Rural village	51 (54.8%)	20 (21.5%)	18 (19.4%)	4 (4.3%)		
Grade: year one	69 (53.1%)	34 (26.2%)	24 (18.5%)	3 (2.3%)	0.905	0.905
Grade: year three	71 (51.1%)	32 (23.0%)	32 (23.0%)	4 (2.9%)		
Grade: year two	85 (54.5%)	37 (23.7%)	28 (17.9%)	6 (3.8%)		
Work status: not working	218 (54.0%)	99 (24.5%)	75 (18.6%)	12 (3.0%)	0.046	0.082
Work status: working	7 (33.3%)	4 (19.0%)	9 (42.9%)	1 (4.8%)		
Health-related condition: No	183 (61.2%)	66 (22.1%)	43 (14.4%)	7 (2.3%)	<0.001	<0.001
Health-related condition: Yes	42 (33.3%)	37 (29.4%)	41 (32.5%)	6 (4.8%)		
Family history of mental illness/depression: No	218 (55.6%)	97 (24.7%)	69 (17.6%)	8 (2.0%)	<0.001	<0.001
Family history of mental illness/depression: Yes	7 (21.2%)	6 (18.2%)	15 (45.5%)	5 (15.2%)		
Smoking: No	222 (55.4%)	99 (24.7%)	72 (18.0%)	8 (2.0%)	<0.001	<0.001
Smoking: Yes	3 (12.5%)	4 (16.7%)	12 (50.0%)	5 (20.8%)		

Percentages are row percentages. Age was tested with Kruskal–Wallis; categorical variables with chi-square. *BH*, Benjamini–Hochberg correction across the nine sociodemographic/health comparisons.

### 3.4 Psychosocial factors across composite score bands

Bullying, sexual harassment, emotional or physical violence, family conflict, and dissatisfaction with physical appearance were each associated with higher composite-score bands after correction for multiple comparisons. Loss of a loved one was not significantly associated with score-band distribution after correction (Table 5).

**Table 5:** Psychosocial exposures across composite score bands.

Exposure/level	Low *n* (%)	Moderate *n* (%)	High *n* (%)	Extreme *n* (%)	Uncorrected *p*	BH-adjusted *p*
Bullying: No	186 (60.4%)	70 (22.7%)	44 (14.3%)	8 (2.6%)	<0.001	<0.001
Bullying: Yes	39 (33.3%)	33 (28.2%)	40 (34.2%)	5 (4.3%)		
Sexual harassment: No	223 (54.9%)	99 (24.4%)	75 (18.5%)	9 (2.2%)	<0.001	<0.001
Sexual harassment: Yes	2 (10.5%)	4 (21.1%)	9 (47.4%)	4 (21.1%)		
Emotional/physical violence: No	209 (62.0%)	77 (22.8%)	46 (13.6%)	5 (1.5%)	<0.001	<0.001
Emotional/physical violence: Yes	16 (18.2%)	26 (29.5%)	38 (43.2%)	8 (9.1%)		
Loss of a loved one: No	100 (61.0%)	34 (20.7%)	26 (15.9%)	4 (2.4%)	0.072	0.072
Loss of a loved one: Yes	125 (47.9%)	69 (26.4%)	58 (22.2%)	9 (3.4%)		
Family conflict: No	216 (60.8%)	83 (23.4%)	51 (14.4%)	5 (1.4%)	<0.001	<0.001
Family conflict: Yes	9 (12.9%)	20 (28.6%)	33 (47.1%)	8 (11.4%)		
Dissatisfaction with physical appearance: No	192 (57.0%)	86 (25.5%)	53 (15.7%)	6 (1.8%)	<0.001	<0.001
Dissatisfaction with physical appearance: Yes	33 (37.5%)	17 (19.3%)	31 (35.2%)	7 (8.0%)		

Percentages are row percentages. *P*-values are from chi-square tests. BH correction was applied across the six psychosocial comparisons.

### 3.5 Factors associated with an elevated composite score

In the 425-student multivariable model, family conflict (aOR = 5.47, 95% CI: 2.52–11.89) and emotional or physical violence (aOR = 3.40, 95% CI: 1.76–6.56) remained significantly associated with an elevated composite score after Benjamini–Hochberg correction (corrected *p* < 0.001 for both). Bullying showed a positive association at the uncorrected threshold (aOR = 1.68, 95% CI: 1.00–2.82, *p* = 0.049) but did not survive correction (corrected *p* = 0.098) and is therefore interpreted as suggestive. Sexual harassment, loss of a loved one, and dissatisfaction with physical appearance were not independently significant in the overall model (Table 6).

**Table 6:** Multivariable logistic regression for elevated composite score (≥25 vs. 0–24), overall and by gender.

Model/predictor	aOR	95% CI	Uncorrected *p*	BH-adjusted *p*	Interpretation after BH
Overall (*n* = 425)					
Bullying	1.68	1.00–2.82	0.049	0.098	Suggestive only
Sexual harassment	3.60	0.67–19.34	0.136	0.170	Not significant
Emotional/physical violence	3.40	1.76–6.56	<0.001	<0.001	Significant
Loss of a loved one	1.39	0.90–2.16	0.141	0.170	Not significant
Family conflict	5.47	2.52–11.89	<0.001	<0.001	Significant
Dissatisfaction with physical appearance	1.33	0.76–2.33	0.313	0.313	Not significant
Male (*n* = 205)					
Bullying	2.08	1.02–4.25	0.044	0.088	Suggestive only
Sexual harassment	0.22	0.02–3.08	0.263	0.395	Not significant
Emotional/physical violence	2.52	1.04–6.10	0.040	0.088	Suggestive only
Loss of a loved one	1.04	0.55–1.96	0.906	0.906	Not significant
Family conflict	5.63	1.80–17.62	0.003	0.018	Significant
Dissatisfaction with physical appearance	0.92	0.43–1.97	0.837	0.906	Not significant
Female (*n* = 220)					
Bullying	1.31	0.59–2.92	0.507	0.507	Not significant
Sexual harassment	10.56	1.12–99.88	0.040	0.080	Suggestive only
Emotional/physical violence	5.45	1.90–15.60	0.002	0.005	Significant
Loss of a loved one	1.88	0.98–3.62	0.057	0.086	Not significant
Family conflict	7.30	2.24–23.77	<0.001	0.005	Significant
Dissatisfaction with physical appearance	2.20	0.93–5.17	0.071	0.086	Not significant

All models contain the same six psychosocial predictors. Overall-model VIF range=1.20–1.80. BH correction was applied across the six predictor *p*-values within each model.

Gender-stratified analyses showed a similar pattern with some differences in effect size. Among male students (*n* = 205), family conflict remained significantly associated with elevated scores after correction (aOR = 5.63, corrected p = 0.018); bullying (aOR = 2.08) and emotional/physical violence (aOR = 2.52) met the uncorrected *p* < 0.05 threshold but did not survive correction (corrected *p* = 0.088 for both). Among female students (*n* = 220), emotional/physical violence (aOR = 5.45, corrected *p* = 0.005) and family conflict (aOR = 7.30, corrected *p* = 0.005) remained significant after correction. Sexual harassment had a large point estimate (aOR = 10.56, 95% CI: 1.12–99.88) but a very wide confidence interval and did not survive correction (corrected *p* = 0.080), so this result is considered suggestive rather than confirmed (Table 6).

### 3.6 Teachers' perspectives on student depressive symptoms

Forty-two teachers from eight secondary schools participated in the qualitative component. Thematic analysis identified five interconnected themes: (1) behavioral and emotional indicators of student depressive symptoms, (2) impact on academic engagement and classroom participation, (3) effects on the broader classroom environment, (4) barriers to identification and support, and (5) recommended interventions.

#### 3.6.1 Theme 1: behavioral and emotional indicators

Teachers consistently identified withdrawal, isolation, and emotional instability as the most recognizable signs of student depressive symptoms. One teacher described the typical presentation in detail:

“First, withdrawing into himself. Other behaviors include sitting at the back of the class, trying to isolate himself from the group, frequently sleeping in class, not paying attention to lessons, and finally, not completing homework or assigned activities at home.”

Physical signs such as weight loss and neglect of personal appearance were also reported, alongside emotional instability characterized by one teacher as a fluctuating mood state “often dominated by playing the role of being sad.”

#### 3.6.2 Theme 2: impact on academic engagement

Teachers reported that depressive symptoms most visibly impaired students' motivation and willingness to participate, rather than their grades directly. One teacher explained:

“Depression makes the student give up and reduces their motivation to learn, feeling that no matter how much effort they put in, they will not understand.”

Subjects most commonly identified as affected included mathematics, science, and English, while extracurricular activities such as school radio, competitions, and physical education were described as the first activities abandoned. As one teacher noted:

“When a student faces depression, it negatively reflects in their lack of response and participation in classroom activities, making them view such activities as unimportant.”

#### 3.6.3 Theme 3: effects on the classroom environment

Several teachers described how a student's depressive state could affect the broader classroom dynamic. One teacher provided a particularly vivid account:

“High school students are very easily influenced by the energy around them. For example, if an active student comes, everyone becomes active with him — and if a student comes in with depression, everyone is affected, and you feel the whole class is depressed.”

Other teachers noted that depressed students could create an atmosphere of fear or discomfort among peers, or impede cooperative learning activities.

#### 3.6.4 Theme 4: barriers to identification and support

The near-universal absence of formal mental health training was a defining finding. The majority of teachers explicitly reported receiving no relevant training, with one stating:

“We have no psychological expertise or special training courses that enable us to identify students suffering from depression and its symptoms.”

Several described their knowledge as informally acquired, as reflected in one teacher's remark: “No courses, but life has taught me.”

Structural barriers were also prominent, particularly large class sizes, as one teacher explained:

“A teacher can recognize a student with a problem if class sizes are small, but if they are large, the teacher should observe students and their changes.”

Parental denial emerged as the most consistently cited obstacle to supporting affected students. Teachers described parents who refused to acknowledge their child's condition, with one noting that families refuse for fear of it spreading among the community. Another captured the cultural dimension more broadly: “Many still believe these cases should remain hidden.” This stigma-driven concealment was perceived as directly impeding referral and early intervention.

#### 3.6.5 Theme 5: recommended interventions

Teachers advocated for systemic, multi-level responses. The most commonly recommended interventions included the placement of a trained psychologist within each school, mandatory mental health training for teachers, and structured family engagement programs. One teacher summarized the overarching need:

“There must be a partnership between the school, the home, and mental health services.”

Practical suggestions included “organizing campaigns inside the school in coordination with the Ministry of Health to screen for depression cases” and “therapy sessions with a psychologist at the end of each month.” Several teachers called explicitly on the Ministry of Education to formalize psychological training as part of teacher preparation.

These qualitative findings complemented and contextualized the quantitative results. Teachers' observations of withdrawal, isolation, and disengagement provided school-level context for the observed associations between psychosocial adversity and elevated composite scores. The barriers identified—particularly parental denial, stigma, and limited formal training—highlight systemic gaps that may impede recognition and referral in school settings (Table 7).

**Table 7:** Summary of qualitative themes from teacher interviews (*n* = 42).

Theme	Key observations	Representative quotation
1. Behavioral and emotional indicators	Withdrawal, isolation, sleeping in class, poor attention, emotional instability, and neglect of appearance.	“First, withdrawing into himself … frequently sleeping in class, not paying attention to lessons.”
2. Academic engagement	Reduced motivation, participation, and extracurricular involvement; perceived effects often preceded grade changes.	“Depression makes the student give up and reduces their motivation to learn.”
3. Classroom environment	Teachers perceived spillover effects on peer mood, cooperation, and classroom climate.	“If a student comes in with depression, everyone is affected.”
4. Barriers to identification and support	Limited formal mental-health training, large classes, parental denial, and stigma impeded recognition and referral.	“We have no psychological expertise or special training courses …”
5. Recommended interventions	School psychologists, teacher training, family engagement, and school–health-service partnerships were repeatedly recommended.	“There must be a partnership between the school, the home, and mental health services.”

### 3.7 Reliability of the depressive-symptom-risk composite

The internal consistency of the 17-item depressive-symptom-risk composite was good in the 425-student analytical sample (Cronbach's *α* = 0.838). Corrected item–total correlations ranged from 0.254 to 0.654 (Table 1).

### 3.8 Depressive symptoms and academic performance

No significant correlation was found between composite scores and CGPA (Spearman's *ρ* = −0.031, *p* = 0.519). Median CGPA values did not differ significantly across the four score bands (Kruskal–Wallis *H* = 0.386, *p* = 0.943; Table 8).

**Table 8:** Composite scores and academic performance (CGPA).

Analysis/score band	*n*	CGPA median (IQR)	Statistic; *p*-value
Low	225	99.00 (4.00)	*H* = 0.386; *p* = 0.943
Moderate	103	99.00 (4.00)	
High	84	98.07 (3.11)	
Extreme	13	98.00 (2.00)	
Spearman correlation (score vs. CGPA)	425	—	*ρ* = −0.031; *p* = 0.519

## 4 Discussion

This mixed-methods study found that nearly half of secondary school students in Bisha Province had an elevated score (≥25) on the study's depressive-symptom-risk/general-distress composite. Because the composite and its empirical score bands are not validated diagnostic thresholds, this proportion should not be interpreted as the prevalence of major depressive disorder. Nonetheless, the high burden of distress-related indicators can be considered alongside Saudi studies reporting substantial but variable adolescent depressive symptoms using validated depression instruments (13, 15, 19–21). International evidence likewise documents a substantial adolescent depression burden across settings (18, 22, 23).

### 4.1 Psychosocial factors associated with elevated composite scores

The regression analysis identified family conflict and emotional or physical violence as the most robust correlates of an elevated composite score after multiple-comparison correction; bullying showed a weaker, suggestive association. Family conflict emerged as the strongest correlate in the overall model. This pattern is consistent with a recent meta-analysis showing a positive association between parent–adolescent conflict and depressive mood (16). Within the Saudi social context, persistent family conflict may plausibly undermine emotional security and perceived support. Teachers' qualitative accounts (Theme 1, Section 3.6.1) also described withdrawal and emotional disturbance in students experiencing family-related difficulties, providing contextual convergence with the survey finding (44).

Bullying was associated with an elevated composite score at the uncorrected significance threshold, although the association did not survive correction for multiple comparisons and should therefore be interpreted as suggestive. This direction is consistent with systematic-review evidence linking bullying victimization to depression, anxiety, suicidal ideation, and other adverse psychosocial outcomes (17, 41, 42), as well as Saudi adolescent evidence connecting bullying and physical violence with poorer mental-health outcomes (24). Teachers likewise described social withdrawal and reduced classroom participation among distressed students (Theme 3, Section 3.6.3), which provides contextual support without establishing causality.

Emotional or physical violence remained significantly associated with elevated scores overall and among female students. This is consistent with Saudi adolescent studies reporting associations of physical and emotional abuse or violence with depression-related outcomes (13, 15, 24). Among female students, sexual harassment had a large point estimate but a very wide confidence interval and did not survive multiple-comparison correction; this should be treated as hypothesis-generating. The direction is nevertheless compatible with evidence from female secondary-school students in Buraydah, where sexual harassment and violence were associated with depression, and with regional evidence on gender differences in adverse childhood experiences and psychological distress (15, 25).

It is also important to note that these relationships are plausibly bidirectional. For example, while family conflict may exacerbate depressive symptoms, adolescents experiencing depressive symptoms may in turn become more withdrawn, irritable, or emotionally reactive in ways that increase friction within the family system; a similar bidirectional pattern may apply to bullying, insofar as socially withdrawn or dysphoric adolescents may become more vulnerable to victimization. The cross-sectional design of this study cannot disentangle the direction of these effects, and the associations reported here should not be interpreted as evidence of causal, unidirectional pathways.

### 4.2 Sociodemographic and health-related correlates

In bivariate analyses, health-related conditions, smoking, and family history of mental illness were associated with higher composite-score bands. These findings should be interpreted cautiously because these variables were not included in the primary six-exposure psychosocial regression model. They are broadly compatible with literature linking depression and anxiety with chronic-condition burden (26) and smoking with depression-related risk in young people and young adults (27, 45). In this sample, gender and grade level were not associated with score-band distribution after correction; this null pattern should not be generalized, as other adolescent studies have reported gender differences in depression and symptom trajectories (13, 28).

### 4.3 Depressive symptoms and academic performance

Although depressive symptoms and distress are often associated with academic difficulties, this study found no significant correlation between composite score and CGPA (Spearman's *ρ* = −0.031, *p* = 0.519). Prior literature indicates that the relationship between depressive symptoms, academic strain, and achievement is heterogeneous and may vary by design and outcome measure (5, 7, 29). CGPA may be relatively insensitive to early changes in motivation, participation, or attendance, particularly in a cross-sectional analysis.

Importantly, the qualitative data offer a meaningful interpretation of this null finding. Teachers consistently described depressed students as experiencing marked declines in motivation and classroom participation before any measurable change in formal grades occurred. As one teacher explained: “Depression makes the student give up and reduces their motivation to learn, feeling that no matter how much effort they put in, they will not understand” [translated from Arabic]. Mathematics, English, and science were identified as the subjects most affected, alongside extracurricular activities. This pattern suggests that engagement and motivation may be more sensitive early indicators of depression's academic impact than cumulative grades, which may be temporarily preserved through compensatory mechanisms such as parental academic pressure or exam-focused remedial support. CGPA, as an aggregate measure, may therefore lack the sensitivity to detect the early functional consequences of depressive symptoms. Future longitudinal studies should incorporate more granular outcome measures — including attendance rates, teacher-assessed participation, and standardized test performance — to more accurately capture the evolving academic impact of depressive symptoms over time.

### 4.4 Teachers' perceptions and qualitative insights

The qualitative findings provide important contextual depth to the quantitative results. Teachers across multiple schools described withdrawal, isolation, emotional instability, and reduced engagement among distressed students. These observations are compatible with literature on how adolescents and families identify and seek help for depression (30) and with studies of school-based mental-health literacy (31).

A critical finding was the near-universal absence of formal mental-health training among participating teachers. Although teachers could often recognize behavioral changes, most reported no structured preparation to distinguish clinically significant mental-health problems from typical adolescent behavior or to initiate appropriate referral. This gap between observational capacity and formal preparedness is consistent with literature on teachers' perceived roles, efficacy, and barriers in supporting student mental health (32, 33), as well as regional work on school health professionals' mental-health literacy (31). Large class sizes were also repeatedly cited as limiting teachers' ability to monitor individual students.

Parental denial and social stigma were identified as persistent barriers to supporting affected students. Teachers described families who were reluctant to acknowledge a child's mental-health difficulty because of perceived social consequences. This is consistent with literature on adolescent and family help-seeking barriers (30, 34). Such barriers may impede early identification and referral, underscoring the importance of family engagement and culturally appropriate destigmatization alongside school-based services.

Teachers' recommendations for school-based psychologists, mental-health training, and structured partnerships between schools, families, and health services are consistent with WHO/UNESCO health-promoting-school guidance and standards (35, 36) and provide a contextually grounded basis for policy development in the Saudi setting.

## 5 Implications for policy and practice

These findings support the need for multi-level, coordinated interventions across school, family, and health-system domains (46). Left unaddressed, clinically significant adolescent depression can carry risks that extend well beyond short-term academic disengagement. Clinical evidence links untreated depressive disorders in children and adolescents with substance misuse, impaired social and later occupational functioning, suicidal behavior, and persistence of depressive illness into adulthood (4). Although the present composite does not diagnose depression, its association with violence and family conflict identifies groups in whom validated assessment and timely support may be particularly important.

The Saudi Ministry of Education should consider integrating validated, age-appropriate mental-health screening into routine school-health programs, accompanied by clear consent, referral, and safeguarding pathways, as well as teacher training in mental-health first aid and symptom recognition (36, 37). The existing school-counselor infrastructure provides a foundation that could be strengthened through psychologist support and defined referral pathways to community mental-health services. Family-centered destigmatization programs are also important given the parental denial reported by teachers. Such multi-tiered approaches are consistent with the WHO Health-Promoting Schools framework (36) and with Saudi Arabia's Vision 2030 emphasis on health, education, and quality of life (38).

## 6 Strengths and limitations

Several methodological strengths should be noted. The convergent mixed-methods design allowed quantitative findings to be contextualized through teachers' classroom observations. The 425-student analytical sample was recruited through stratified multistage cluster sampling across boys' and girls' schools, and the balanced sex composition supported gender-stratified analyses. Recalculation of all reported quantitative analyses on the stated 425-student sample ensured consistency after exclusion of the age outlier. The primary multivariable model used a common six-exposure psychosocial specification in overall and gender-stratified analyses, with Benjamini–Hochberg correction and explicit multicollinearity assessment. The qualitative component included 42 teacher interviews and provided contextual insight into school manifestations of distress and barriers to support.

Several limitations should be acknowledged. First, the cross-sectional design precludes causal inference between psychosocial factors and composite scores and cannot rule out bidirectional relationships (see Section 4.1). Second, reliance on self-reported data introduces possible recall and social-desirability bias, particularly for sensitive exposures. Third, the 17-item composite uses 0–7 response coding (theoretical total 0–119) and primarily assesses emotional suppression, somatic complaints, aggression/irritability, risk-taking behavior, feeling unsupported, and emotion-control difficulty. It does not directly assess both DSM-5 core symptoms of persistent dysphoria and anhedonia, nor sleep, appetite, or suicidal ideation, and it does not closely reproduce the PHQ-9 or CES-D (39, 40). The empirical score bands (0–24, 25–38, 39–67, and ≥68) are sample-specific and have not been externally validated against diagnostic interviews; therefore, the reported 47.1% with scores ≥25 is an elevated-composite-score proportion, not a clinical depression prevalence estimate. Fourth, the study was conducted in one province, limiting generalizability to the broader Saudi adolescent population and particularly to major metropolitan settings such as Riyadh and Jeddah. Fifth, no clinical diagnostic interviews were conducted. Sixth, electronic administration through WhatsApp groups may have underrepresented students without reliable smartphone access. Finally, the qualitative teacher sample was voluntary and non-probability-based, the exact interview at which saturation was first reached was not prospectively recorded, and interviews were conducted in Arabic and translated for reporting, so some nuance may have been lost in translation.

## 7 Conclusion

This study found that 47.1% of secondary school students in Bisha Province had an elevated score (≥25) on the study's depressive-symptom-risk/general-distress composite. Family conflict and exposure to emotional or physical violence were the most robust psychosocial correlates after correction for multiple comparisons; bullying and, among female students, sexual harassment showed positive but statistically suggestive associations warranting further study. Qualitative findings from 42 teachers showed recognizable behavioral and academic changes among distressed students, while highlighting limited formal training, parental denial, and stigma as barriers to support. With appropriate caution regarding the non-diagnostic composite and the cross-sectional design, the findings support the use of validated school-based mental-health assessment, evidence-based anti-bullying and violence-prevention strategies, structured teacher training, and family-centered awareness and referral programs in Saudi Arabia.

## Data Availability

The original contributions presented in the study are included in the article/Supplementary Material, further inquiries can be directed to the corresponding author.
