# Supplementary material for: Prevalence and psychosocial correlates of depressive symptom risk among secondary school students in Saudi Arabia: a mixed-methods study
Source: Front Child Adolesc Psychiatry. 2026 Sep 2;5:1926540. doi: 10.3389/frcha.2026.1926540 (PMC13578071; doi:10.3389/frcha.2026.1926540)
Supplement: Supplementary file 1 [file Table1.docx]

**Supplementary Table 2. Teachers’ Perspectives on Student Depression and Support Needs (N = 42)**

| **Domain / Theme** | **Response category** | **n** | **%** |
| --- | --- | --- | --- |
| **Observed changes in students’ mental health** | No change observed | 9 | 21.4 |
|  | Change observed | 8 | 19.0 |
|  | Emotional and behavioral changes | 7 | 16.7 |
|  | Social and communication issues | 7 | 16.7 |
|  | Academic stress and low motivation | 4 | 9.5 |
|  | Technology and social media influence | 3 | 7.1 |
|  | Other observations* | 4 | 9.5 |
| **Distinguishing depression from normal adolescence** | No change observed | 10 | 23.8 |
|  | Social withdrawal and isolation | 10 | 23.8 |
|  | Change observed | 8 | 19.0 |
|  | Lack of interest in studies | 5 | 11.9 |
|  | Normal developmental changes | 5 | 11.9 |
|  | Environmental and social influences | 4 | 9.6 |
| **Impact on academic and extracurricular performance** | Overall academic decline | 13 | 31.0 |
|  | Reduced motivation and engagement | 5 | 11.9 |
|  | Extracurricular difficulties | 5 | 11.9 |
|  | Subject-specific difficulties | 6 | 14.3 |
|  | Concentration and comprehension problems | 3 | 7.1 |
|  | No effect observed | 5 | 11.9 |
| **Classroom behavior during depressive episodes** | Withdrawal and isolation | 13 | 31.0 |
|  | Reduced classroom engagement | 11 | 26.2 |
|  | No change observed | 5 | 11.9 |
|  | Emotional distress or instability | 6 | 14.2 |
|  | Fatigue and low energy | 2 | 4.8 |
| **Effect on classroom environment** | Negative class atmosphere | 11 | 26.2 |
|  | Reduced interaction and participation | 9 | 21.4 |
|  | Isolation of affected student | 8 | 19.0 |
|  | Limited or no impact | 8 | 19.0 |
| **Support strategies used or recommended** | Academic and classroom support | 10 | 23.8 |
|  | Psychological and emotional support | 10 | 23.8 |
|  | Family involvement | 6 | 14.3 |
|  | Supportive classroom environment | 5 | 11.9 |
|  | Addressing root causes | 4 | 9.5 |
|  | Other approaches† | 7 | 16.7 |
| **Training received by teachers** | No formal training | 17 | 40.5 |
|  | Psychology/counseling-related training | 11 | 26.2 |
|  | General educational training | 4 | 9.5 |
|  | Informal or self-learning | 9 | 21.4 |
| **Challenges in detection and support** | Overcrowding and workload | 10 | 23.8 |
|  | Student-related barriers | 10 | 23.8 |
|  | Parental or societal denial | 7 | 16.7 |
|  | Lack of training or knowledge | 6 | 14.3 |
|  | Institutional barriers | 3 | 7.2 |
| **Communication difficulties with parents** | Poor cooperation or communication | 11 | 26.2 |
|  | Lack of awareness | 10 | 23.8 |
|  | Parental denial | 9 | 21.4 |
|  | Stigma-related concerns | 6 | 14.3 |
| **Recommended awareness strategies** | Awareness programs | 15 | 35.7 |
|  | Supportive school environment | 9 | 21.4 |
|  | Strengthening counseling services | 8 | 19.0 |
|  | Teacher training | 5 | 11.9 |
| **Single most important intervention** | Psychological counseling | 15 | 35.7 |
|  | Recreational activities | 7 | 16.7 |
|  | Individual teacher–student support | 4 | 9.5 |
|  | Institutional improvements | 4 | 9.5 |
|  | Other responses‡ | 12 | 28.6 |

**Footnotes:**
Responses from 12 open-ended questions were grouped into major themes based on shared content.

**Abbreviations:** N, number of respondents.

* Includes autism, weight loss, family or societal pressure, and positive change.
† Includes extracurricular activities, religious guidance, and school counseling.
‡ Includes no clear response, awareness initiatives, parental involvement, and community collaboration.
