# Supplementary material for: Prevalence and psychosocial correlates of depressive symptom risk among secondary school students in Saudi Arabia: a mixed-methods study
Source: Front Child Adolesc Psychiatry. 2026 Sep 2;5:1926540. doi: 10.3389/frcha.2026.1926540 (PMC13578071; doi:10.3389/frcha.2026.1926540)
Supplement: Supplementary file 2 [file Table2.docx]

| **Analysis** | **Measure** | **Value** |
| --- | --- | --- |
| **Depression score (continuous)** | Spearman correlation coefficient (*r*) | −0.031 |
|  | *p* value | 0.518 |
| **Depression risk category** |  |  |
| Low | n = 226 | Median CGPA = 39.0 |
| Moderate | n = 103 | Median CGPA = 87.0 |
| High | n = 84 | Median CGPA = 74.8 |
| Extreme | n = 13 | Median CGPA = 94.0 |

**Supplementary Table 4. Association between Depression and Academic Performance (CGPA, %)**
