# Supplementary material for: Prevalence and psychosocial correlates of depressive symptom risk among secondary school students in Saudi Arabia: a mixed-methods study
Source: Front Child Adolesc Psychiatry. 2026 Sep 2;5:1926540. doi: 10.3389/frcha.2026.1926540 (PMC13578071; doi:10.3389/frcha.2026.1926540)
Supplement: Supplementary file 3 [file Table3.docx]

**Supplementary Table 1. Overview of Open-Ended Questions Exploring Teachers’ Perspectives on Student Depression (N = 42)**

| **Question ID** | **Focus of question** |
| --- | --- |
| Q1 | Observed changes in students’ mental health over time |
| Q2 | Distinguishing normal adolescent behavior from symptoms of depression |
| Q3 | Impact of depression on students’ academic and extracurricular performance |
| Q4 | Behavioral changes in classroom participation during depressive episodes |
| Q5 | Effect of a depressed student on classroom environment and peer interaction |
| Q6 | Effective methods for supporting students with depression |
| Q7 | Training received for identifying and managing student depression |
| Q8 | Challenges in detecting and supporting depressed students |
| Q9 | Difficulties in communicating with parents of depressed students |
| Q10 | Advice for teachers on recognizing and supporting students with psychological difficulties |
| Q11 | Recommended strategies to raise mental health awareness in schools |
| Q12 | Single most important intervention to improve depression management in schools |

**Footnote:**
This table summarizes the thematic focus of the 12 open-ended questions administered to participating school teachers (N = 42).
