# Supplementary material for: Prevalence and psychosocial correlates of depressive symptom risk among secondary school students in Saudi Arabia: a mixed-methods study
Source: Front Child Adolesc Psychiatry. 2026 Sep 2;5:1926540. doi: 10.3389/frcha.2026.1926540 (PMC13578071; doi:10.3389/frcha.2026.1926540)
Supplement: Supplementary file 4 [file Table4.docx]

**Supplementary Table 3. Reliability Analysis of the Depression Risk Scale (Item–Total Statistics)**

| **Item** | **Corrected Item–Total Correlation** | **Cronbach’s Alpha if Item Deleted** |
| --- | --- | --- |
| I ignored bad emotions. | 0.321 | 0.839 |
| I hid my problems. | 0.519 | 0.825 |
| I smoke more than usual. | 0.348 | 0.835 |
| I drove recklessly or violently. | 0.301 | 0.835 |
| My heartburn was more than average. | 0.497 | 0.826 |
| I had regular headaches. | 0.506 | 0.825 |
| My stomach hurts. | 0.482 | 0.827 |
| I was left to figure things out on my own. | 0.539 | 0.823 |
| I experienced strange aches and sensations. | 0.654 | 0.815 |
| I need to smoke to help me relax. | 0.390 | 0.833 |
| I need to have easy access to cigarettes. | 0.312 | 0.835 |
| I reacted excessively and aggressively to circumstances. | 0.495 | 0.826 |
| I stopped worrying about the results of my conduct. | 0.254 | 0.840 |
| I took unnecessary dangers. | 0.574 | 0.822 |
| I attempted to ignore feeling depressed. | 0.451 | 0.829 |
| I was verbally aggressive against others. | 0.451 | 0.829 |
| It was tough to control my emotions. | 0.518 | 0.824 |

**Overall Cronbach’s alpha (17 items):** 0.837

**Footnotes:**
Internal consistency was assessed using Cronbach’s alpha
