# Supplementary material for: Prevalence and psychosocial correlates of depressive symptom risk among secondary school students in Saudi Arabia: a mixed-methods study
Source: Front Child Adolesc Psychiatry. 2026 Sep 2;5:1926540. doi: 10.3389/frcha.2026.1926540 (PMC13578071; doi:10.3389/frcha.2026.1926540)
Supplement: Supplementary file 5 [file Table5.docx]

**Tables:**

| **Table 1 Sociodemographic characteristics of participants** | | | |
| --- | --- | --- | --- |
| **Age (years)** | | | |
| Minimum | 13 | | |
| Maximum | 19 | | |
| Normality | Statistic | | 0.855 |
|  | *P*-value | | <0.001 |
|  | Median (IQR) | | 17 (2) |
| **Study variables** | | ***n* (%)** | |
| **Gender** | | | |
| Males | | 205 (48.1) | |
| Females | | 220 (51.8) | |
| **Living status** | | | |
| Living with my family | | 372 (87.6) | |
| Living with my father only | | 7 (1.6) | |
| Living with my mother only | | 43 (10.1) | |
| Living with my aunt | | 1 (0.2) | |
| Living with my grandmother | | 1 (0.2) | |
| Living alone | | 1 (0.2) | |
| **Education level** | | | |
| Year one | | 130 (30.5) | |
| Year two | | 156 (36.6) | |
| Year three | | 139 (32.9) | |
| **Work status** | | | |
| Working | | 21 (4.9) | |
| Not working | | 404 (95.1) | |
| **Place of residency** | | | |
| Bisha city | | 332 (78.2) | |
| Rural village | | 93 (21.8) | |
| **Health-related issue** | | | |
| Yes | | 127 (29.8) | |
| No | | 298 (70.2) | |
| **Family history of mental illness or depression** | | | |
| Yes | | 33 (7.7) | |
| No | | 392 (92.3) | |
| **Smoking** | | | |
| Yes | | 24 (5.6) | |
| No | | 401 (94.4) | |
| Normality of age was tested by the Shapiro-Wilk test. Health-related issues included cardiovascular and metabolic disorders, respiratory disorders, neurological and psychiatric disorders, gastrointestinal disorders, renal stones, thyroid disease, anemia, lipomas, dermatological diseases, ophthalmological and ENT (Ear, Nose, Throat) disorders, dental problems, surgical and injury-related conditions. **Abbreviations:** IQR, interquartile range. | | | |

| **Table 2 Reliability analysis of the depression risk scale (Item–total statistics)** | | |
| --- | --- | --- |
| **Study questions to evaluate depression** | **Corrected Item-Total Correlation** | **Cronbach's Alpha** |
| I ignored bad emotions. | 0.321 | 0.839 |
| I hid my problems. | 0.519 | 0.825 |
| I smoke more than usual. | 0.348 | 0.835 |
| I drove recklessly or violently. | 0.301 | 0.835 |
| My heartburn was more than average. | 0.497 | 0.826 |
| I had regular headaches. | 0.506 | 0.825 |
| My stomach hurts. | 0.482 | 0.827 |
| I was left to figure things out on my own. | 0.539 | 0.823 |
| I experienced strange aches and sensations. | 0.654 | 0.815 |
| I need to smoke to help me relax. | 0.390 | 0.833 |
| I need to have easy access to cigarettes. | 0.312 | 0.835 |
| I reacted excessively and aggressively to circumstances. | 0.495 | 0.826 |
| I stopped worrying about the results of my conduct. | 0.254 | 0.840 |
| I took unnecessary dangers. | 0.574 | 0.822 |
| I attempted to ignore feeling depressed. I verbally attack the others without being provoked. | 0.451 | 0.829 |
| I was verbally aggressive against others. | 0.451 | 0.829 |
| It was tough to control my emotions. | 0.518 | 0.824 |
| **Reliability Statistics (Cronbach's Alppha for all the 17 items)** | | 0.837 |
| Reliability analysis was done considering Cronbach’s Alpha values. | | |

| **Table 3 Prevalence of depression among study participants** | | | | |
| --- | --- | --- | --- | --- |
| **Descriptive statistics** | | | | |
| Cumulative depression score | **Minimum** | **Maximum** | **Mean** | **Standard deviation** |
|  | 0 | 84 | 25.70 | 19.1 |
| **Distribution of participants in categories of depression** | | | | |
| **Depression risk** | **Number of participants (%)** | | | |
| Low | 226 (53) | | | |
| Moderate | 102 (24) | | | |
| High | 84 (20) | | | |
| Extreme | 13 (3) | | | |
| Low risk scores 0–24, i.e. ≤ 20% of 119, moderate risk: 25–38, i.e. 21–32% of 119, high risk: 39–67, i.e. 33–56% of 119, and extreme risk: 68–119, i.e. 57–100%. The total study participants was 426. | | | | |

| **Table 4 Correlation between depression and academic performance (CGPA/%)** | | | | | | | | |
| --- | --- | --- | --- | --- | --- | --- | --- | --- |
| **CGPA/%** | | **Normality test** | | | | | | |
|  |  | Statistic | | | | 0.602 | | |
|  |  | *p*-value | | | | <0.001 | | |
|  |  | Median (IQR) | | | | 99 (4) | | |
| **Depression as continuous scores** | | | | | | | | |
| **Correlation coefficient** | | -0.031 | | | | | | |
| ***p*-value** | | 0.518 | | | | | | |
| **Depression as categories** | | | | | | | | |
| **Descriptive analysis** | | | | | **Kruskal-Wallis test** | | | |
| **Categories** | ***n*** | | **Minimum** | **Maximum** | **Mean rank** | **Kruskal-Wallis H value** | ***p*-value** | |
| Low risk | 226 | | 39.00 | 100.00 | 215.77 | 0.395 | | 0.941 |
| Moderate risk | 103 | | 87.00 | 100.00 | 214.46 |  |  |  |
| High risk | 84 | | 74.80 | 100.00 | 208.21 |  |  |  |
| Extreme risk | 13 | | 94.00 | 100.00 | 200.62 |  |  |  |
| Normality of CGPA/% was tested by the Shapiro-Wilk test. The correlation between depression (as continuous scores 0 to 119) and academic performance was evaluated using Spearman’s rho correlation test. However, comparison of CGPA/% for different categories of depression was evaluated via the Kruskal-Wallis test. | | | | | | | | |

| **Table 5 Comparison of sociodemographic characteristics between students with low depression risk and students with moderate-to-extreme depression risk** | | | | | | |
| --- | --- | --- | --- | --- | --- | --- |
| **Variables** | **Response** | **Low depression risk (*n* = 226)** | **Moderate-to-extreme depression risk (*n* = 200)** | **Mann-Whitney U / Pearson Chi-Square** | ***p*-value** | ***p*-value _(corrected)_** |
| **Age (years)** | Minimum | 14 | 25 | 21812.5**^a^** | 0.517 | - |
|  | Maximum | 13 | 18 |  |  |  |
|  | Mean rank | 210.02 | 217.44 |  |  |  |
| **Gender** | Males | 102 (45.1%) | 103 (51.5%) | 1.723 | 0.189 | 0.2205 |
|  | Females | 124 (54.9%) | 97 (48.5%) |  |  |  |
| **Living status** | Living with my family | 207 (91.6%) | 166 (83.0%) | 8.833 | **0.012** | **0.012** |
|  | Living with one parent | 19 (8.4%) | 31 (15.5%) |  |  |  |
|  | Living with relatives/alone | 0 (0.0%) | 3 (1.5%) |  |  |  |
| **Education level** | Year one | 69 (30.5%) | 61 (30.5%) | 0.277 | 0.871 | 0.871 |
|  | Year two | 85 (37.6%) | 71 (35.5%) |  |  |  |
|  | Year three | 72 (31.9%) | 68 (34.0%) |  |  |  |
| **Work status** | Working | 7 (3.1%) | 14 (7.0%) | 3.448 | 0.063 | 0.0882 |
|  | Not working | 219 (96.9%) | 186 (93.0%) |  |  |  |
| **Health-related issue** | Yes | 43 (19.0%) | 84 (42.0%) | 26.762 | **< 0.001** | **< 0.001** |
|  | No | 183 (81.0%) | 116 (58.0%) |  |  |  |
| **Family history of mental illness or depression** | Yes | 7 (3.1%) | 26 (13.0%) | 14.559 | **0.0001** | **0.0002** |
|  | No | 219 (96.9%) | 174 (87.0%) |  |  |  |
| **Smoking** | Yes | 3 (1.3%) | 21 (10.5%) | 16.792 | **< 0.001** | **< 0.001** |
|  | No | 223 (98.7%) | 179 (89.5%) |  |  |  |
| The study participants were categorized into low depression risk and moderate-to-extreme depression risk. Age was compared between two groups by Mann-Whitney U Test. All the categorical variables were compared between groups by Chi-Square test. Health-related issues included cardiovascular and metabolic disorders, respiratory disorders, neurological and psychiatric disorders, gastrointestinal disorders, renal stones, thyroid disease, anemia, lipomas, dermatological diseases, ophthalmological and ENT (Ear, Nose, Throat) disorders, dental problems, surgical and injury-related conditions. Age is specified with Mann-Whitney U value and *p*-value**^a^**. Living with one parent included living with mother or father. In the response category living with relatives/alone, relatives include grandmother or aunt. | | | | | | |

| **Table 6 Risk factors associated with depression** | | | | | | | | |
| --- | --- | --- | --- | --- | --- | --- | --- | --- |
| **Risk factors** | **Response** | **Depression risk** | | | | | | |
|  |  | **Low** | **Elevated** | **High** | **Extreme** | ***χ*² value** | ***p*-value** | ***p*-value _(corrected)_** |
| Being bullied | Yes | 39 (33.3%) | 33 (28.2%) | 40 (34.2%) | 5 (4.3%) | 30.82 | **< 0.001** | **< 0.001** |
|  | No | 187 (60.5%) | 70 (22.7%) | 44 (14.2%) | 8 (2.6%) |  |  |  |
| Sexual harassment | Yes | 2 (10.5%) | 4 (21.1%) | 9 (47.4%) | 4 (21.1%) | 35.69 | **< 0.001** | **< 0.001** |
|  | No | 224 (55.0%) | 99 (24.3%) | 75 (18.4%) | 9 (2.2%) |  |  |  |
| Emotional/physical violence | Yes | 16 (18.2%) | 26 (29.5%) | 38 (43.2%) | 8 (9.1%) | 70.96 | **< 0.001** | **< 0.001** |
|  | No | 210 (62.1%) | 77 (22.8%) | 46 (13.6%) | 5 (1.5%) |  |  |  |
| Lost a loved one | Yes | 126 (48.1%) | 69 (26.3%) | 58 (22.1%) | 9 (3.4%) | 6.81 | 0.078 | 0.078 |
|  | No | 100 (61.0%) | 34 (20.7%) | 26 (15.9%) | 4 (2.4%) |  |  |  |
| Family conflicts | Yes | 9 (12.9%) | 20 (28.6%) | 33 (47.1%) | 8 (11.4%) | 77.39 | **< 0.001** | **< 0.001** |
|  | No | 217 (61.0%) | 83 (23.3%) | 51 (14.3%) | 5 (1.4%) |  |  |  |
| Satisfaction with physical appearance | Yes | 192 (57.0%) | 86 (25.5%) | 53 (15.7%) | 6 (1.8%) | 27.45 | **< 0.001** | **< 0.001** |
|  | No | 34 (38.2%) | 17 (19.1%) | 31 (34.8%) | 7 (7.9%) |  |  |  |
| The association of risk factors with depression was examined by the Pearson Chi-Square Test of Independence (*χ*²). Loved one, including any family member, relative, or friend. | | | | | | | | |

| **Table 7 Binary logistic regression analysis of psychosocial risk factors associated with depression among secondary-school students** | | | | | | | |
| --- | --- | --- | --- | --- | --- | --- | --- |
| **Risk factors** | **Response** | **Depression risk** | | **OR** | **95% C.I** | ***p*-value** | ***p*-value _(corrected)_** |
|  |  | **Low** | **Moderate-extreme** |  |  |  |  |
| Being bullied | Yes | 39 (33.3%) | 78 (66.7%) | 1.696 | 1.011-2.845 | 0.045 | 0.09 |
|  | No | 187 (60.5%) | 122 (39.5%) |  |  |  |  |
| Sexual harassment | Yes | 2 (10.5%) | 17 (89.5%) | 3.613 | 0.672-19.438 | 0.135 | 0.18 |
|  | No | 224 (55.0%) | 183 (45.0%) |  |  |  |  |
| Emotional/physical violence | Yes | 16 (18.2%) | 72 (81.8%) | 3.422 | 1.775-6.599 | 0.0002 | **0.0006** |
|  | No | 210 (62.1%) | 128 (37.9%) |  |  |  |  |
| Lost a loved one | Yes | 126 (48.1%) | 136 (51.9%) | 1.380 | 0.890-2.140 | 0.150 | 0.18 |
|  | No | 100 (61.0%) | 64 (39.0%) |  |  |  |  |
| Family conflicts | Yes | 9 (12.9%) | 61 (87.1%) | 5.500 | 2.531-11.950 | 0.00002 | **0.00012** |
|  | No | 217 (61.0%) | 139 (39.0%) |  |  |  |  |
| Satisfaction with physical appearance | No | 34 (38.2%) | 55 (61.8%) | 0.771 | 0.443-1.344 | 0.359 | 0.359 |
|  | Yes | 192 (57.0%) | 145 (43.0%) |  |  |  |  |
| **Males** | | | | | | | |
| Being bullied | Yes | 18 (31.0%) | 40 (69.0%) | 2.082 | 1.020-4.249 | 0.044 | 0.088 |
|  | No | 84 (57.1%) | 63 (42.9%) |  |  |  |  |
| Sexual harassment | Yes | 1 (25.0%) | 3 (75.0%) | 0.223 | 0.016-3.083 | 0.263 | 0.395 |
|  | No | 101 (50.2%) | 100 (49.8%) |  |  |  |  |
| Emotional/physical violence | Yes | 10 (23.8%) | 32 (76.2%) | 2.523 | 1.043-6.102 | 0.040 | 0.088 |
|  | No | 92 (56.4%) | 71 (43.6%) |  |  |  |  |
| Lost a loved one | Yes | 64 (46.7%) | 73 (53.3%) | 1.039 | 0.552-1.955 | 0.906 | 0.906 |
|  | No | 38 (55.9%) | 30 (44.1%) |  |  |  |  |
| Family conflicts | Yes | 5 (15.6%) | 27 (84.4%) | 5.634 | 1.801-17.622 | 0.003 | **0.018** |
|  | No | 97 (56.1%) | 76 (43.9%) |  |  |  |  |
| Satisfaction with physical appearance | No | 19 (44.2%) | 24 (55.8%) | 1.083 | 0.507-2.314 | 0.837 | 0.906 |
|  | Yes | 83 (51.2%) | 79 (48.8%) |  |  |  |  |
| **Females** | | | | | | | |
| Being bullied | Yes | 21 (35.6%) | 38 (64.4%) | 1.341 | 0.604-2.975 | 0.471 | 0.471 |
|  | No | 103 (63.6%) | 59 (36.4%) |  |  |  |  |
| Sexual harassment | Yes | 1 (6.7%) | 14 (93.3%) | 10.609 | 1.125-100.063 | 0.039 | 0.078 |
|  | No | 123 (59.7%) | 83 (40.3%) |  |  |  |  |
| Emotional/physical violence | Yes | 6 (13.0%) | 40 (87.0%) | 5.530 | 1.933-15.820 | 0.001 | **0.003** |
|  | No | 118 (67.4%) | 57 (32.6%) |  |  |  |  |
| Lost a loved one | Yes | 62 (49.6%) | 63 (50.4%) | 1.838 | 0.959-3.522 | 0.067 | 0.101 |
|  | No | 62 (64.6%) | 34 (35.4%) |  |  |  |  |
| Family conflicts | Yes | 4 (10.5%) | 34 (89.5%) | 7.318 | 0.250-23.800 | 0.001 | **0.003** |
|  | No | 120 (65.6%) | 63 (34.4%) |  |  |  |  |
| Satisfaction with physical appearance | No | 15 (32.6%) | 31 (67.4%) | 0.492 | 0.212-1.141 | 0.098 | 0.118 |
|  | Yes | 109 (62.3%) | 66 (37.7%) |  |  |  |  |
| The frequencies of the risk factor categories are presented as counts and percentages. The association between different risk factors and depression risk was sought by binary logistic regression. To identify the most predictive risk factors of depression among secondary school students, the association of each factor was sought while adjusting for other factors. The *p*-values are provided. A *p*-value < 0.05 is considered statistically significant. The *p*-values were corrected for multiple comparisons by Benjamin–Hochberg method. Statistically significant and corrected *p*-values are shown in **bold**. Odd ratios and 95% confidence intervals were calculated to estimate the associated risk. Abbreviations: OR odds ratio, CI confidence interval, loved one, including any family member, relative, or friend. | | | | | | | |

| **Table 8 Teachers’ perspectives on student depression to improve support and well-being** | | |
| --- | --- | --- |
| **Responses’ categories/themes** | **Frequency of responses** | **Percentage** |
| **Total number of respondents (N) = 42** | | |
| **Observed changes in students’ mental health over time** | | |
| No change observed | 9 | 21.4 |
| Change observed | 8 | 19.0 |
| Emotional and behavioral changes | 7 | **16.7** |
| Social and communication issues | 7 | **16.7** |
| Academic stress and low motivation | 4 | 9.5 |
| Technology and social media influence | 3 | 7.1 |
| Autism | 1 | 2.4 |
| Weight loss | 1 | 2.4 |
| Family/societal pressure | 1 | 2.4 |
| Positive change | 1 | 2.4 |
| **Distinguishing normal adolescent behavior from symptoms of depression** | | |
| No change observed | 10 | 23.8 |
| Social withdrawal and isolation | 10 | **23.8** |
| Change observed | 8 | 19.0 |
| Lack of interest in studies | 5 | 11.9 |
| Normal adolescent developmental changes | 5 | 11.9 |
| Influence of social and environmental factors | 3 | 7.1 |
| Seriousness for study | 1 | 2.4 |
| **Impact of depression on students’ academic and extracurricular performance** | | |
| Negative impact of depression on overall academic performance | 13 | **31.0** |
| Impact on extracurricular and group activities | 5 | 11.9 |
| Lack of motivation and disinterest in learning | 5 | 11.9 |
| Subject-specific difficulties, Science subjects. | 4 | 9.5 |
| Reduced concentration, focus, and comprehension | 3 | 7.1 |
| Social and emotional withdrawal | 3 | 7.1 |
| Effects observed | 2 | 4.8 |
| Stress from exams and high expectations | 2 | 4.8 |
| No effect observed | 2 | 4.8 |
| No effect observed | 1 | 2.4 |
| Subject-specific difficulties, mathematics. | 1 | 2.4 |
| Subject-specific difficulties, Religious and Arabic subjects. | 1 | 2.4 |
| **Behavioral changes in classroom participation during depression episodes** | | |
| Withdrawal and Isolation | 13 | **31.0** |
| Reduced Classroom Engagement | 11 | **26.2** |
| No changes observed | 5 | 11.9 |
| Changes observed | 4 | 9.5 |
| Emotional Distress and Crying | 3 | 7.1 |
| Emotional Instability and Aggression | 3 | 7.1 |
| Fatigue and Low Energy | 2 | 4.8 |
| Cognitive and Behavioral Changes | 1 | 2.4 |
| **Effect of a depressed student on classroom environment and interaction** | | |
| Negative Influence on Class Atmosphere | 11 | **26.2** |
| Decreased Classroom Interaction and Participation | 9 | **21.4** |
| Isolation and Withdrawal | 8 | 19.0 |
| Limited or No Impact on Others | 5 | 11.9 |
| Multiple effects | 3 | 7.1 |
| No effect | 3 | 7.1 |
| Potential Positive or Mixed Effects | 2 | 4.8 |
| Need for Special Attention and Handling | 1 | 2.4 |
| **Effective methods for supporting students with depression** | | |
| Academic Support and Classroom Inclusion | 10 | **23.8** |
| Psychological and Emotional Support | 10 | **23.8** |
| Family Involvement and Home-School Cooperation | 6 | 14.3 |
| Creating Supportive Classroom Environment | 5 | 11.9 |
| Identifying and Addressing Root Causes | 4 | 9.5 |
| Extracurricular and Physical Activities | 2 | 4.8 |
| Religious and Moral Guidance | 2 | 4.8 |
| School-Based Counseling and Teacher Involvement | 2 | 4.8 |
| No response | 1 | 2.4 |
| **Training received for identifying and managing student depression** | | |
| No Formal Training | 17 | **40.5** |
| Psychology and Counseling-Related Courses | 11 | **26.2** |
| General Educational and Behavioral Training | 4 | 9.5 |
| Life Experience and Informal Learning | 4 | 9.5 |
| Self-Learning and Personal Experience | 3 | 7.1 |
| Indirectly Related Training | 2 | 4.8 |
| Expression of Need for Specialized Training | 1 | 2.4 |
| **Challenges in detecting and supporting depressed students** | | |
| Overcrowded classrooms and workload | 10 | **23.8** |
| Student-related barriers | 10 | **23.8** |
| Parental and societal denial | 7 | 16.7 |
| Lack of training and knowledge | 6 | 14.3 |
| Student absenteeism and disengagement | 3 | 7.1 |
| Difficulty distinguishing depression from normal behavior | 2 | 4.8 |
| Lack of institutional and structural support | 2 | 4.8 |
| No answer | 1 | 2.4 |
| Reliance on referral system | 1 | 2.4 |
| **Difficulties in communicating with parents of depressed students** | | |
| Lack of cooperation and poor communication | 11 | **26.2** |
| Lack of parental awareness and understanding | 10 | **23.8** |
| Parental denial and refusal to acknowledge depression | 9 | 21.4 |
| Stigma and social reputation concerns | 6 | 14.3 |
| No answer | 3 | 7.1 |
| Family issues affecting communication | 2 | 4.8 |
| Lack of conviction or seriousness | 1 | 2.4 |
| **Advice for teachers on recognizing and supporting students with psychological issues** | | |
| Empathy, patience, and supportive attitude | 10 | **23.8** |
| Administrative and systemic support | 9 | **21.4** |
| Communication and dialogue | 6 | 14.3 |
| Observation and monitoring of student behavior | 6 | 14.3 |
| Awareness and preventive education | 3 | 7.1 |
| Education and training for teachers | 3 | 7.1 |
| Encouraging inclusion and engagement | 2 | 4.8 |
| None | 3 | 7.1 |
| **Recommended strategies to raise mental health awareness in schools** | | |
| Awareness programs and educational campaigns | 15 | **35.7** |
| Creating a supportive and engaging school environment | 9 | 21.4 |
| Strengthening the role of school counselors and psychologists | 8 | 19.0 |
| Training and capacity building for teachers and staff | 5 | 11.9 |
| Collaboration between school, family, and external institutions | 3 | 7.1 |
| None | 2 | 4.8 |
| **Single most important intervention to improve depression management in schools** | | |
| Psychological counseling and professional support | 15 | **35.7** |
| No clear response | 7 | 16.7 |
| Recreational and extracurricular activities | 7 | 16.7 |
| Individual attention and teacher-student interaction | 4 | 9.5 |
| Institutional or structural improvements | 4 | 9.5 |
| Religious or moral guidance | 2 | 4.8 |
| Awareness and parental involvement | 2 | 4.8 |
| Collaboration and community-based interventions | 1 | 2.4 |
| The responses of 12 open-ended questions were categorized into major themes according to key ideas. | | |

| **Supplementary Table 1 Understanding teachers’ perspectives on student depression to improve support and well-being** | |
| --- | --- |
| **Question ID** | **Question** |
| Q1 | Observed changes in students’ mental health over the time |
| Q2 | Distinguishing normal adolescent behavior from symptoms of depression |
| Q3 | Impact of depression on students’ academic and extracurricular performance |
| Q4 | Behavioral changes in classroom participation during depression episodes |
| Q5 | Effect of a depressed student on classroom environment and interaction |
| Q6 | Effective methods for supporting students with depression |
| Q7 | Training received for identifying and managing student depression |
| Q8 | Challenges in detecting and supporting depressed students |
| Q9 | Difficulties in communicating with parents of depressed students |
| Q10 | Advice for teachers on recognizing and supporting students with psychological issues |
| Q11 | Recommended strategies to raise mental health awareness in schools |
| Q12 | Single most important intervention to improve depression management in schools |
| This table includes the major idea of each question asked from respondents (school teachers) of the survey. Total 12 questions were asked from a total number of 42 respondents. | |
